# Liver-Related COVID-19 Consequences: Dynamics of Liver Health in 2.5 Years

**DOI:** 10.3390/jcm14217604

**Published:** 2025-10-27

**Authors:** Ieva Vanaga, Oksana Kolesova, Aleksandrs Kolesovs, Maija Radzina, Davis Simanis Putrins, Jelena Egle, Sniedze Laivacuma, Jelena Storozenko, Ludmila Viksna

**Affiliations:** 1 Department of Infectology, Riga Stradins University, 3 Linezera Street, LV-1006 Riga, Latvia; ieva.vanaga@rsu.lv (I.V.);; 2 Riga East University Hospital, 2 Hipokrata Street, LV-1079 Riga, Latvia; 3 Institute of Microbiology and Virology, Riga Stradins University, 5 Ratsupites Street, LV-1067 Riga, Latvia; 4 Radiology Department, Riga Stradins University, 16 Dzirciema Street, LV-1007 Riga, Latvia; 5 Faculty of Health and Life Sciences, University of Latvia, 3 Jelgavas Street, LV-1004 Riga, Latvia; 6 Institute of Diagnostic Radiology, Pauls Stradins Clinical University Hospital, LV-1002 Riga, Latvia

**Keywords:** liver fibrosis, liver injury, steatosis, COVID-19, post-COVID

## Abstract

**Objectives:** This study aimed to assess the dynamics of liver tests (LT) and detect signs of liver fibrosis and steatosis 2.5 years after the first COVID-19 episode in patients without pre-existing liver-related conditions. **Methods:** The study included 65 adult patients hospitalized with COVID-19 (including 18 with severe or critical illness) in 2020. After 2.5 years, in addition to regular LT, liver health status was assessed by the FIB-4 index, hyaluronic acid, cytokeratin 18 fragment M30 (serum, ELISA), cardiometabolic risk factors, and the multiparametric ultrasound examination. **Results:** LT abnormalities in the acute COVID-19 period were observed more frequently (*p* = 0.036) in patients with severe or critical COVID-19 (83%) than in patients with non-severe COVID-19 (55%). LT dynamics in 2.5 years showed an improvement of liver health status in most patients (*p* = 0.006). Persistent LT abnormalities were associated with LT abnormalities during hospitalization (*p* = 0.021). After 2.5 years, the presence of cardiometabolic risk factors and signs of liver fibrosis were associated with the severity of the first COVID-19 episode. However, regression analyses did not support disease severity as a predictor for LT abnormalities and liver stiffness. The latter was predicted by cardiovascular diseases in the anamnesis. **Conclusions:** In most patients, LT normalized despite potential risk factors. Simultaneously, in some patients, signs of liver fibrosis after COVID-19 might be stimulated by COVID-19-related metabolic dysfunction and the presence of cardiovascular diseases.

## 1. Introduction

Long-term liver injury and fibrosis were previously mentioned as significant COVID-19 consequences [1,2,3,4,5]. Immunological, metabolic, and endothelial dysfunctions caused by the SARS-CoV-2 virus during acute COVID-19 and possibly persisting after clinical recovery can trigger Kupffer cells and hepatic stellate cells (HSC). It can lead to enhanced production and accumulation of extracellular matrix (ECM) components in the liver [1,3]. Additionally, the liver could be affected by behavior changes due to social isolation and restrictions in the COVID-19 pandemic [3,6]. Increasing alcohol use, reduced physical activity, and excessive caloric intake, associated with higher self-reported anxiety and depression, could predispose to the development of steatotic liver disease (SLD) [3,6,7,8]. Moreover, adverse effects on the liver might be enhanced by SARS-CoV-2 reinfection [9] and COVID-19 vaccination [10]. All mentioned factors can negatively contribute to liver health globally [3,6,7,8].

Currently, COVID-19-related liver injury and fibrosis are insufficiently explored from a long-term perspective. In the acute illness, liver injuries were mainly mild [11,12]. However, its prevalence varied across the studies [13,14,15] due to the lack of universally accepted or standardized criteria for defining liver injury in COVID-19. Previous longitudinal studies [16,17,18] demonstrated the positive dynamics of liver tests (LT) within six [18], 12 [16], and 20 months [17] after COVID-19. In these studies, older age [18], male gender, high BMI [16], hospitalization [17], and post-COVID syndrome [18] were mentioned as factors associated with residual clinical and biochemical alterations of the liver. However, mechanisms for persistent LT abnormalities in some patients remain underexplored.

Our previous studies [19,20] demonstrated the increased liver fibrogenesis and the presence of hepatic steatosis 3–9 months after acute COVID-19 in some patients. Therefore, the present study aimed to assess the dynamics of LT in a more extended period than previous follow-ups [16,17,18] and detect signs of liver fibrosis and steatosis 2.5 years after the first COVID-19 episode. Based on previous reviews and studies [1,2,3,4,5,16,17,18,19,20], we can expect the persistence of LT abnormalities for a longer than 20-month period, which can be associated with the first COVID-19 severity. In addition, we can expect that signs of liver fibrosis at follow-up can be related to the severity of the first-time COVID-19. To test these assumptions, the study group was divided into two subgroups according to the severity of the first-time COVID-19 episode. To avoid complications during the biopsy, multiparametric ultrasound (mpUS) was used with non-invasive liver fibrosis tests such as FIB-4 [21,22]. Considering that patients after COVID-19 are at risk for developing liver fibrosis [1,2,3,4,5], we assessed lifestyle and metabolic factors in the cohort at follow-up that can affect liver health status [21,22]. Additional information regarding liver health status was obtained by detection of markers of liver disease in stored plasma samples [23] such as hyaluronic acid (HA)—the main component of ECM produced by fibroblasts and specialized connective tissue cells [24], and cytokeratin 18 (CK18) fragment M30 (CK18-M30), which increases in sera after caspase-cleaved hepatocyte apoptosis [25].

## 2. Materials and Methods

### 2.1. Study Design

The Ethics Committee of Riga Stradins University, Latvia (protocol No. 6-1/07/14 and protocol No. 4/354/2022) approved the study. All participants signed the informed consent form, and the study followed the Declaration of Helsinki.

A longitudinal study was conducted between 2020 and 2024 and included 101 adult patients hospitalized with a first-time COVID-19 episode from September to December 2020, before the routine COVID-19 vaccination started in Latvia after January 2021. SARS-CoV-2 infection was confirmed by reverse transcription polymerase chain reaction (RT-PCR) in a nasopharyngeal swab. Patients with viral hepatitis (HCV, HBV, and HAV), HIV infection, autoimmune hepatitis, haemochromatosis, Wilson’s disease, and hepatocellular carcinoma, as well as pregnant women, were excluded from the study. Two patients died in the acute period. All survivors were invited to participate in the follow-up about 2.5 years after the discharge.

### 2.2. Baseline Assessment

The baseline assessment was performed on data obtained from medical documentation and a questionnaire. It included social demographic data, comorbidities, characterization of COVID-19 severity, routine clinical tests, including liver tests (LT)—alanine aminotransferase (ALT) and/or aspartate aminotransferase (AST) and/or γ-glutamyltransferase (GGT) and/or alkaline phosphatase (ALP) at admission to the hospital and during hospitalization. Diagnostic examinations of liver images were not performed for epidemiological safety and availability reasons.

LT abnormalities were defined as one or more liver enzyme values exceeding the upper limit of normal value (ULN) according to reference laboratory standards: ALT level (male > 40 U/L, female > 35 U/L); AST level (male > 40 U/L, female > 32 U/L); GGT level (male > 60 U/L, female > 40 U/L); ALP level (male > 129 U/L, female > 104 U/L) [13,15]. Additionally, LT abnormalities were categorized based on the degree of liver enzyme elevation as mild (1–2 times of ULN), moderate (>2–5 times of ULN), and severe (>5 times of ULN) [14,26].

Based on the severity of COVID-19, as non-severe, severe, and critical, defined according to the World Health Organization classification [27], patients were divided into two groups: patients with non-severe COVID-19 and patients with severe or critical COVID-19 episodes.

### 2.3. The Follow-Up Assessment

The follow-up assessment focused on more detailed liver function and possible metabolic factor assessment using biochemical parameters in serum and liver tissue assessment by mpUS. During the follow-up, all patients underwent an extended investigation of their health status assessed through face-to-face visits lasting around 30–45 min, including a certified hepatologist’s physical examination. In addition, peripheral blood samples were obtained on an empty stomach and ALT, AST, GGT, ALP, total and direct bilirubin, lactate dehydrogenase (LDH), prothrombin index (PI), D-dimers, C-reactive protein (CRP), triglycerides (TG), and high-density lipoprotein (HDL) cholesterol.

During the visit, all patients answered survey questions regarding their subjective recovery, new or long-lasting (over three months) symptoms 2.5 years after the first COVID-19 episode, possible reinfections with SARS-CoV-2 after the hospitalization, SARS-CoV-2 vaccination status, and lifestyle-related factors such as smoking, alcohol use by the AUDIT-C test [28], daily physical activity habits assessed by measuring their weekly duration [29].

For all patients at follow-up, cardiometabolic factors and the index of liver fibrosis (FIB-4) were assessed [17]. FIB-4 was calculated based on formula [30]: FIB-4 = [Age (years) × AST (U/L)]/[PLT (×103/µL) × [ALT1/2 (U/L)]], and categorized in three groups [22]: (1) <1.3; (2) 1.3–2.67; (3) >2.67.

After obtaining mpUS data, suspected metabolic dysfunction-associated steatotic liver disease (MASLD) was defined [22].

### 2.4. mpUS at Follow-Up

The ultrasound examination was performed in a minimum of 3 h of fasting by two certified radiologists. Protocol included 2D shear-wave elastography (2D SWE) for quantitative analysis of liver stiffness and presence of fibrosis in kPa and shear wave dispersion pattern (SWD) in (m/s)/kHz for detection of liver tissue viscosity alteration related to the presence of inflammation. The quantitative evaluation of liver steatosis was performed by Attenuation Imaging (ATI) and measured in dB/cm/MHz with Aplio i800 of Canon Medical Systems equipment (Tochigi, Japan).

All three parameters were obtained in a selected homogenous area of the liver parenchyma with at least five consequent measurements taken to obtain median values in the supine position from the right liver lobe. Measurements were considered reliable if the interquartile range/median ratio (IQR/M) was lower than 0.3. In addition, hepatic attenuation imaging (ATI gen) was performed, and the attenuation coefficient (dB/cm/MHz) was calculated to assess the presence and quantify steatosis.

The cut-off value for minimal fibrosis (F1) was defined starting at 7.1 kP [31].

The presence of hepatic steatosis was defined if ATI ≥ 0.63 dB/cm/MHz [22].

### 2.5. Markers of Liver Disease

Peripheral blood samples were obtained during the first 48 h after admission to the hospital for most of the hospitalized patients and at follow-up for all patients. Blood serum was obtained by centrifugation of peripheral blood samples (1500 rpm) for 20 min at room temperature, then frozen at −80 °C until assay. HA and CK18-M30 were detected by enzyme-linked immunosorbent assay kits (Hyaluronan Quantikine ELISA Kit, R&D Systems, Minneapolis, MN, USA, and M30 Apoptosense ELISA Kit, Peviva, Nacka, Sweden, respectively) following the manufacturer’s instructions.

### 2.6. Statistical Analysis

Data analysis was performed using the SPSS 22.0 statistical package (IBM, New York, NY, USA). Normality of distribution was assessed by the Shapiro–Wilk test. For continuous variables, we performed a *t*-test and a Mann–Whitney test for group comparisons. Differences in categorical variables were detected using the chi-squared method or two-tailed Fisher’s exact test for independent groups. We applied the McNemar test for related samples to assess the dynamics of a categorical variable. Univariate and multivariate regression analyses were used to evaluate predictive models. Linear and logistic regressions were applied to predict continuous and binary outcomes. For all analyses, a two-tailed *p*-value < 0.05 was considered statistically significant. Effect sizes were evaluated by Cohen’s d for the *t*-test, r for the Mann–Whitney test, and Cramer’s V for the chi-square test. The odds ratio (OR) was calculated to compare the presence of LT abnormalities in groups by severity at the baseline, and a paired OR was calculated to assess the dynamics of LT abnormalities. All evaluations of the effect size included a 95% confidence interval (CI). Nagelkerke R^2^ and R^2^ represented effects for logistic and linear regression, respectively.

## 3. Results

### 3.1. Baseline Characteristics

The prospective study included 65 patients with the first COVID-19 episode: 47 (72%) had non-severe COVID-19 and 18 (28%) had severe or critical COVID-19 disease course. Table 1 presents the differences between groups by COVID-19 severity. Patients did not differ between groups in age. The main differences included a higher BMI (*p* < 0.001, Cohen’s d = 1.05, 95% CI [0.47; 1.62]), more frequent comorbidities (*p* = 0.017, Cramer’s V = 0.30, 95% CI [0.05; 0.54]), especially cardiovascular diseases (*p* = 0.014, Cramer’s V = 0.30, 95% CI [0.06; 0.55]), and male sex (*p* = 0.014, Cramer’s V = 0.30, 95% CI [0.06; 0.55]) for patients with severe or critical COVID-19 than for patients with non-severe COVID-19. Observed differences in the admission to ICU (*p* = 0.019, Cramer’s V = 0.34, 95% CI [0.10; 0.58]) and pneumonia (*p* = 0.006, Cramer’s V = 0.36, 95% CI [0.11; 0.60]) were in accordance with the assessment of the COVID-19 severity. Treatment of patients with severe COVID-19 included a higher count of drugs (*p* < 0.001, Cohen’s d = 1.23, 95% CI [0.65; 1.81]), including corticosteroids (*p* < 0.001, Cramer’s V = 0.48, 95% CI [0.24; 0.73]), than treatment of patients with non-severe COVID-19 during the first episode.

### 3.2. LT Abnormalities in the Acute COVID-19

Different degrees of LT abnormalities in the acute COVID-19 period were observed in 15 (83%) patients with severe or critical COVID-19 and 26 (55%) patients with non-severe COVID-19. Among them, 3 (17%) patients and 4 (8.5%) patients had severe LT abnormalities. Mild LT abnormalities were present in 5 (28%) and 10 (21%) patients in severe or critical and non-severe COVID-19, respectively. There was no difference between groups regarding the distribution of degrees of LT abnormalities (*p* = 0.203). However, normal LT vs. Abnormal LT were more often presented in patients with non-severe COVID-19 than in patients with severe or critical COVID-19 (*p* = 0.036). The relative risk of abnormal LT for patients with severe or critical COVID-19 was 4.04 times higher than those with non-severe COVID-19 (OR = 4.04, 95% CI [1.03; 15.84]; *p* = 0.045).

Among liver enzymes, more complete information was obtained for ALT and its dynamics during hospitalization. In contrast, the AST level was detected irregularly. Therefore, the FIB-4 index was not assessed in acute COVID-19. Patients with severe or critical COVID-19 had a higher ALT level during hospitalization than patients with non-severe COVID-19 (*p* = 0.002, r = 0.38, 95% CI [0.15; 0.61]). In addition, HA was higher in patients with severe and critical COVID-19 than in patients with non-severe disease course (*p* = 0.019, r = 0.36, 95% CI [0.07; 0.64]).

### 3.3. Health Status After 2.5 Years

Most patients (83% in the non-severe group and 94% in the severe COVID-19 group) reported different symptoms, which lasted more than three months after discharge from the hospital. It points to a possible development of post-COVID-19 condition. Table 2 presents differences between groups regarding the first COVID-19 severity in metabolic risk factors, routine clinical tests, markers of liver disease, mpUS data, and self-reported data 2.5 years after discharge.

After 2.5 years, patients with a severe or critical first COVID-19 episode had a higher BMI (*p* < 0.001, Cohen’s d = 1.09, 95% CI [0.51; 1.66]), higher TG (*p* = 0.042, r = 0.25, 95% CI [0.01; 0.49]), and lower HDL-cholesterol (*p* = 0.033, r = 0.26, 95% CI [0.03; 0.50]) level than patients with non-severe COVID-19 in anamnesis. However, patients with BMI ≥ 25 kg/m^2^ were presented similarly in both groups (*p* = 0.173).

Assessment of other cardiometabolic risk factors associated with MASLD did not differ between groups. Similarly, there were no differences in liver enzymes, other routine clinical tests, and markers of liver disease. HA and CK18-M30 levels significantly decreased (*p* < 0.001) 2.5 years after hospitalization.

Based on FIB-4, 17 (36%) of patients with a non-severe COVID-19 episode and 8 (44%) patients with severe or critical COVID-19 might be at risk of advanced liver fibrosis or liver-related outcomes. The assessment by the multiparametric US indicated possible liver fibrosis in 4 (8%) patients in the non-severe group and 4 (22%) in severe or critical COVID-19 patients. Likewise, patients with the presence of liver fibrosis by mpUS (median SWE > 7.1 kPa) had a higher FIB-4 (median FIB-4 was 1.52 [IQR: 1.40; 1.72]) than patients without it (median FIB-4 was 1.11 [IQR: 0.76; 1.36]), z = −2.70, *p* = 0.007, r = 0.30, 95% CI (0.07; 0.54).

Analysis of mpUS parameters also showed a higher median SWE after 2.5 years in patients with severe or critical COVID-19 (5.9 kPa [IQR: 5.2; 7.6]) than in patients with non-severe COVID-19 (5.2 kPa [IQR: 4.2; 3.8]), *p* = 0.019. A similar tendency was observed for median ATI; however, it had only marginal significance (*p* = 0.056).

The presence of steatosis was identified in 40% of patients with non-severe and 61% of patients with severe or critical COVID-19 during hospitalization. Suspected MASLD was defined in 8 (17%) and 7 (39%) patients with non-severe and severe or critical first COVID-19 episode, with no statistically significant difference (*p* = 0.100) between groups.

### 3.4. Dynamics of LT

Moderate LT abnormalities after 2.5 years were observed in 2 (11%) patients with severe or critical COVID-19 and 6 (13%) patients with non-severe COVID-19. Mild LT abnormalities were presented in 4 (22%) and 15 (32%) patients in severe or critical and non-severe COVID-19, respectively. There was no difference between groups regarding the degree of liver injury (*p* = 0.693) or the frequency of Normal vs. Abnormal LT (*p* = 0.406).

Analysis of LT dynamics in 2.5 years showed significant improvement of liver health status in most patients regarding the severity of the first COVID-19 episode (McNemar’s χ^2^ = 7.54, *p* = 0.006). Twenty (31%) patients with LT abnormalities in acute COVID-19 did not show them after 2.5 years. However, 6 (9%) patients without LT abnormalities in the acute COVID-19 developed them 2.5 years after discharge. The odds of normalizing LT were 3.33 times higher (95% CI [1.34; 8.30]) than the odds of developing LT abnormalities. Other patients maintained the previous LT status (28% of patients without LT abnormalities, and 32% with them).

A hierarchical logistic regression included LT abnormalities during the first COVID-19 episode and its severity as expected predictors of persistent LT abnormalities at the first step. The results showed that LT abnormalities after 2.5 years were associated with LT abnormalities observed during the first hospitalization with COVID-19, *p* = 0.021. However, there was no direct association with COVID-19 course severity (Table 3). The second step involved age, gender, BMI, and post-COVID condition as previously observed predictors. The step indicated no significant change in the model, χ^2^ = 4.79, *p* = 0.310, and was not included into the table.

A bivariate regression analysis revealed a marginally significant (*p* = 0.052) association between median SWE after 2.5 years and the first COVID-19 severity (R^2^ = 0.06).

For a more detailed analysis, we included factors with significant or marginally significant *p*-levels at baseline and after 2.5 years into hierarchical statistical linear regression. At the baseline, potential predictors were COVID-19 severity, BMI at baseline, comorbidities, cardiovascular diseases, maximal ALT, and abnormal LT during hospitalization. After 2.5 years, they were BMI, TG, HDL-cholesterol, and median ATI. The patient’s age and sex were controlled at the first step of regression. The regression model (Table 4) was significant (*p* < 0.001, R^2^ = 0.23). It included patients’ age (β = 0.38) at the first step, and the presence of cardiovascular diseases (β = 0.34) at the second step. The changing significance of age indicated a possible indirect effect on the median SWE after 2.5 years.

## 4. Discussion

During hospitalization, about half (55%) of patients with non-severe COVID-19 and most patients (83%) with severe or critical disease had LT abnormalities. In the acute period of the illness, LT abnormalities were mainly mild, in line with other studies [11,12]. Excluding pre-existing liver diseases such as chronic viral hepatitis, HIV infection, autoimmune hepatitis, hemochromatosis, Wilson’s disease, and hepatocellular carcinoma allows us to assume an absence of the pathological process in the liver before the first COVID-19 episode and the development of LT abnormalities during hospitalization with acute SARS-CoV-2 infection.

LT dynamics after 2.5 years showed improved liver health status for most patients. This finding is in line with other studies [16,17,18] demonstrating the dynamics of LT within 6 to 20 months after COVID-19. However, we observed that some patients had LT abnormalities after 2.5 years despite normal LT during acute COVID-19, which required a more detailed assessment of liver tissue by mpUS, lifestyle, and metabolic factors.

Despite other studies identifying older age, male gender, high BMI, and post-COVID syndrome as factors associated with persistence of LT abnormalities [16,17,18], we found that LT abnormalities after 2.5 years were more often observed in patients with LT abnormalities during acute COVID-19. It might point to long-term liver injury after COVID-19. However, the regression analysis demonstrated that LT abnormalities after 2.5 years could develop regardless of the severity of the first episode. This finding rejects our hypothesis. Simultaneously, it extends the liver health problem to a broader context than COVID-19 severity.

In this regard, it is important to analyze liver fibrosis as a consequence of COVID-19 [1,2,3]. Considering HA is a non-invasive marker of liver fibrosis [24], differences in its level between patients’ groups in the acute disease confirmed the fibrogenesis stimulated by inflammation. Based on this finding, we could identify patients with severe or critical COVID-19 as having a higher risk for liver fibrosis after COVID-19. Indeed, after 2.5 years, signs of liver fibrosis were confirmed by mpUS in 8% of patients with non-severe and 22% of patients with severe or critical first COVID-19, which was related to the severity of the first COVID-19 episode. We should note that data regarding liver fibrosis prevalence in other post-COVID imaging studies are very limited. In addition to our previous studies on liver fibrosis after COVID-19 [19,20], only one study investigated the liver with two-dimensional shear wave elastography in 2 to 6 months after COVID-19 [32]. This study observed a significant increase in liver stiffness value in the post-COVID infection group (*n* = 34) compared to those who had never been infected (*n* = 34). Moreover, higher SWE values were observed in patients who had a shorter time after COVID-19. However, the association between liver injury and changing liver stiffness remained unclear [32]. To investigate this possible relationship, we performed regression analysis of median SWE after 2.5 years and factors associated with the severity of the first COVID-19 episode and LT abnormalities during hospitalization. The regression analysis did not directly support this association. At the same time, the median SWE after 2.5 years was associated with the presence of cardiovascular diseases in the anamnesis. To extend the view on the revealed link, we assessed the cardiometabolic risk factors included in the MASLD definition [22] and the presence of MASLD in both groups.

MASLD is a common reason for LT abnormalities and liver fibrosis, presented in more than 30% of the population [33,34,35]. In the COVID-19 studies [11,12], MASLD and COVID-19 were frequent co-diagnoses that mutually worsen the course of both diseases [5,11]. Based on our medical documentation data at the baseline, MASLD was documented in less than 10% of patients. A more precise assessment of MASLD after 2.5 years revealed MASLD in 17% of non-severe and 39% of severe or critical patients, which is in line with the negative population trend [33,35]. On the one hand, MASLD may have been underdiagnosed in our cohort before the first COVID-19 episode. On the other hand, it might develop as a post-COVID-19 consequence due to SARS-CoV-2 infection-stimulated metabolic dysfunction [1,5,36,37]. Considering the observed association between a higher BMI and TG level and a lower HDL-cholesterol level in blood after 2.5 years and more severe COVID-19 disease course, we could support COVID-associated metabolic dysfunction in our patients.

Vascular alteration was previously described as a factor that can cause liver fibrosis in acute COVID-19 [38]. Hypoxia-induced factors in COVID-19 can also exacerbate obesity and insulin resistance [39], leading to lipogenesis in the liver and promoting MASLD [39,40,41]. Considering that metabolic dysfunction is a risk factor for cardiovascular diseases [42], we can support the role of cardiometabolic dysfunction in the COVID-related liver fibrosis [1,2,3]. SARS-CoV-2 virus can cause endothelial injury, immune activation, and exacerbate metabolic dysfunction, which are common mechanisms driving fibrosis progression in different organs and cardiovascular pathologies [1,2]. Based on this, we can hypothesize that more aggressive liver fibrosis after COVID-19 occurs in older patients than in younger ones. Monitoring the liver status in older patients using non-invasive tests and mpUS could help start the treatment promptly. It concurs with the suggestion of long-term follow-up studies to explore potential long-term sequelae of SARS-CoV-2 infection [1].

The sample size forms the main limitation of this follow-up study, presenting a unique contribution to studies on liver health in the context of COVID-19. The beginning of the COVID-19 pandemic in Latvia was associated with restricted communication of medical personnel with patients, limiting research participant recruitment. In addition, some clinical tests were unavailable for some patients during the first episode. The sample size decreased from 99 to 65 because of missing data and dropouts within 2.5 years. Consequently, the 95% CIs for all observed effects were relatively wide, and even large and medium estimated effects included medium and small effects, respectively, at their lower limits. It is also possible that patients who experienced negative health consequences adhered to the study to a greater extent. It limits the generalization of findings to people with a mild disease course without further complaints. Epidemiological safety and availability reasons at the end of 2020 limited radiological examination of the liver during the acute COVID-19 episode. Therefore, we analyzed radiological data in a cross-sectional manner.

## 5. Conclusions

The liver health status in patients with excluded pre-existing liver pathology significantly improved 2.5 years after the first COVID-19 episode. In most patients, LT normalized despite potential risk factors. LT abnormalities after 2.5 years were associated with LT abnormalities during hospitalization. The persistence of LT abnormalities and the presence of signs of liver fibrosis were not directly predicted by the severity of the first COVID-19 episode. Observed COVID-19-related metabolic dysfunction and the presence of cardiovascular diseases in the anamnesis might stimulate liver fibrosis after COVID-19 in some patients.

## Figures and Tables

**Table 1:** Baseline characteristics of patients at the first COVID-19 episode.

Variables	COVID-19 Severity		*p*-Value
	Non-Severe *n* = 47	Severe or Critical *n* = 18	
Age, M ± SD, years	50 ± 14	55 ± 12	0.170
Females, *n* (%)	29 (62%)	5 (28%)	**0.014**
BMI, M ± SD, kg/m^2^	29.6 ± 5.9	36.0 ± 6.7	**<0.001**
Smoking, *n* (%)	6 (13%)	5 (28%)	0.161
Comorbidities, *n* (%)	27 (57%)	16 (89%)	**0.017**
Cardiovascular diseases, *n* (%)	17 (38%)	13 (72%)	**0.014**
Chronic respiratory diseases, *n* (%)	3 (6%)	1 (6%)	>0.999
Type 2 diabetes, *n* (%)	5 (11%)	1 (6%)	>0.999
Psychoneurological diseases, *n* (%)	7 (15%)	0	0.176
MASLD, *n* (%)	3 (6%)	2 (11%)	0.611
Thyroid diseases, *n* (%)	5 (11%)	2 (11%)	>0.999
Oncologic diseases, *n* (%)	0	1 (6%)	0.277
Daily medication, *n* (%)	12 (26%)	8 (44%)	0.139
Immunosuppressive drugs, *n* (%)	0	0	NA
**COVID-19 characteristics**			
Pneumonia, *n* (%)	32 (68%)	18 (100%)	**0.006**
Admission to ICU, *n* (%)	0	3 (17%)	**0.019**
**Treatment during hospitalization**			
Mean count of drugs, M ± SD	6 ± 3	9 ± 3	**<0.001**
Most-DILI drugs, *n* (%)	38 (81%)	13 (72%)	0.507
Corticosteroids, *n* (%)	14 (30%)	15 (83%)	**<0.001**
Antibacterial drugs, *n* (%)	38 (81%)	17 (94%)	0.261
Mechanical lung ventilation	0	0	NA
**LT abnormalities**			
Normal LT, *n* (%)	21 (45%)	3 (17%)	0.203 ^a^
Mild, *n*, (%)	10 (21%)	5 (28%)	
Moderate, *n*, (%)	12 (25.5%)	7 (38%)	
Severe, *n* (%)	4 (8.5%)	3 (17%)	
**The highest level of transaminases**			
ALT, Median (IQR), U/L	34 (19; 62)	76 (40; 129)	**0.002**
AST, Median (IQR), U/L	32 (19; 43)	41 (25; 61)	0.153
Missing data, *n*	17	1	
**Markers of liver disease**			
HA, Median (IQR), ng/mL	46.5 (29.2; 82.1)	83.9 (47.9; 284.2)	**0.019**
Missing data, *n*	19	4	
CK18-M30, Median (IQR), U/L	204.0 (124.5; 246.0)	230.0 (205.0; 325.0)	0.159
Missing data, *n*	21	4	

Notes: Titles for subgroups of variables are presented in bold. *n*—number of patients; BMI—body mass index; MASLD—metabolic dysfunction-associated steatotic liver disease; ICU—intensive care unit; DILI—drug-induced liver injury; LT—liver tests; ALT—alanine aminotransferase; AST—aspartate aminotransferase; HA—hyaluronic acid; CK18-M30—cytokeratin 18 fragment M30. Mean (M) and standard deviation (SD) represent normally distributed variables. Median and interquartile range (IQR) represent non-normally distributed variables. Significant *p*-values are presented in bold. ^a^—*p*-value is calculated for the distribution of LT abnormalities in four subgroups (from normal to severe).

**Table 2:** Severity of the first COVID-19 in relation to liver functioning markers, potentially affecting factors, and ultrasound data after 2.5 years.

Variables	COVID-19 Severity		*p*-Value
	Non-Severe *n* = 47	Severe or Critical *n* = 18	
**Self-reported data**			
Reinfection, *n* (%)	14 (30%)	2 (11%)	0.198
Vaccination against SARS-CoV-2, *n* (%)	41 (89%)	15 (83%)	0.676
Suspected post-COVID condition, *n* (%)	39 (83%)	17 (94%)	0.425
No regular physical activity, *n* (%)	30 (64%)	11 (61%)	0.839
Smoking, *n* (%)	3 (6%)	1 (6%)	>0.999
Daily medication, *n* (%)	28 (60%)	13 (72%)	0.344
Statins, *n* (%)	2 (4%)	3 (17%)	0.125
**Metabolic risk factors**			
BMI, M ± SD, kg/m^2^	30.0 ± 5.5	36.6 ± 7.3	**<0.001**
BMI ≥ 25.0 kg/m^2^, *n* (%)	40 (74%)	18 (100%)	0.173
Waist circumference: ≥94 cm for men or ≥80 cm for women, *n* (%)	41 (93%)	18 (100%)	0.550
Dysglycaemia or Type 2 diabetes	5 (11%)	1 (6%)	>0.999
TG ≥ 1.7 mmol/L or lipid lowering treatment, *n* (%)	10 (21%)	6 (33%)	0.346
HDL-cholesterol ≤ 1.0 mmol/L for men; ≤1.3 mmol/L for women or lipid lowering treatment, *n* (%)	12 (26%)	4 (22%)	>0.999
Blood pressure ≥130/85 of hypotensive treatment, *n* (%)	22 (50%)	11 (61%)	0.426
AUDITc ≥ 4 for men; ≥3 for women	17 (36%)	4 (22%)	0.282
**LT abnormalities**			
Normal LT, *n* (%)	26 (55%)	12 (67%)	0.693 ^a^
Mild, *n* (%)	15 (32%)	4 (22%)	
Moderate, *n*, (%)	6 (13%)	2 (11%)	
Severe, *n* (%)	0	0	
**Biochemical parameters**			
ALT, Median (IQR), U/L	25 (19; 36)	32 (21; 42)	0.308
AST, Median (IQR), U/L	28 (22; 37)	26 (22; 33)	0.587
GGT, Median (IQR), U/L	26 (16; 51)	26 (22; 33)	0.814
ALP, Median (IQR), U/L	69 (56; 86)	69 (53; 85)	0.769
LDH, Median (IQR), U/L	180 (158; 203)	193 (183; 206)	0.139
Total bilirubin, Median (IQR), μmol/L	9.1 (6.6; 12.6)	9.2 (8.2; 13.7)	0.305
Direct bilirubin, Median (IQR), μmol/L	4.3 (3.3; 5.9)	4.6 (3.3; 6.2)	0.519
Prothrombin index, Median (IQR), %	102.7 (95.5; 112.5)	102.7 (93.4; 104.7)	0.238
D-dimers Median (IQR), mg/L	0.36 (0.19; 0.56)	0.39 (0.29; 0.67)	0.190
Albumin Median (IQR), g/L	46 (45; 47)	46 (46; 48)	0.730
CRP Median (IQR), mg/L	2.1 (0.7; 2.9)	1.9 (0.6; 5.4)	0.524
TG Median (IQR), mmol/L	1.10 (0.80; 1.54)	1.48 91.18; 1.88)	**0.042**
HDL-cholesterol, Median (IQR), mmol/L	1.42 (1.23; 1.67)	1.23 (1.08; 1.42)	**0.033**
**Markers of liver disease**			
HA, Median (IQR), ng/mL	25.3 (16.4; 37.7)	27.1 (11.2; 38.3)	0.823
Missing data, *n*	1	0	
CK18-M30, Median (IQR), U/L	131.0 (97.4; 148.7)	129.2 (112.2; 160.2)	0.654
FIB-4, Median (IQR)	1.13 (0.68; 1.36)	1.12 (0.76; 1.53)	0.965
<1.3, *n* (%)	30 (64%)	10 (56.0%)	-
1.3–2.67, *n* (%)	16 (34%)	8 (44.0%)	-
≥2.67, *n* (%)	1 (2%)	0	-
**Multiparametric US**			
SWE, Median (IQR), kPa	5.2 (4.3; 5.8)	5.9 (5.2; 7.6)	**0.019**
SWE ≥ 7.1 kPa, *n* (%)	4 (8%)	4 (22%)	-
SWD, Median (IQR), (m/s)/kHz	11.1 (10.1; 12.4)	10.8 (9.7; 12.9)	0.709
Missing data, *n*	2	0	-
ATI, Median (IQR), dB/cm/MHz	0.59 (0.53; 0.69)	0.68 (0.59; 0.73)	0.056
Missing data, *n*	2	0	**-**
≥0.63 dB/cm/MHz, *n* (%)	18 (40%)	11 (61%)	-
**Suspected MASLD, *n* (%)**	8 (17%)	7 (39%)	0.100
Missing data, *n*	1	0	-

Notes: Titles for subgroups of variables are presented in bold. *n*—number of patients; BMI—body mass index; ICU—intensive care unit; AUDITc—Alcohol Use Disorders Identification Test C; LT—liver tests; ALT—alanine aminotransferase; AST—aspartate aminotransferase; GGT—gamma-glutamyl transferase; ALP—alkaline phosphatase; LDH—lactate dehydrogenase; CRP—C-reactive protein; TG—triglycerides; HDL-cholesterol—high-density lipoprotein cholesterol; HA—hyaluronic acid; CK18-M30—cytokeratin 18 fragment M30; FIB-4—liver fibrosis index; SWE—shear-wave elastography; SWD—shear-wave dispersion; ATI—attenuation imaging; MASLD—metabolic dysfunction-associated steatotic liver disease. Mean (M) and standard deviation (SD) represent normally distributed variables. Median and interquartile range (IQR) represent non-normally distributed variables. Significant *p*-values are presented in bold. ^a^—*p*-value is calculated for the distribution of LT abnormalities in four subgroups (from normal to severe).

**Table 3:** Logistic regression predicting LT abnormalities 2.5 years after the first COVID-19.

Variables	B	S.E.	Wald χ^2^	df	*p*	Exp(B)	95% CI for Exp(B)
Constant	−1.01	0.48	4.49	1	**0.034**	0.36	-
COVID-19 severity	−0.89	0.62	2.02	1	0.155	0.41	0.12; 1.40
LT abnormalities	1.38	0.60	5.34	1	**0.021**	3.98	1.23; 12.86

Notes: B—non-standardized regression coefficient; S.E.—standard error; df—degrees of freedom; Exp(B)—odds ratio; LT—liver tests. CI—confidence interval. Significant *p*-values are presented in bold. Nagelkerke R^2^ = 0.13.

**Table 4:** Hierarchical statistical regression predicting the median SWE after 2.5 years (*n* = 62).

Variables		B	S.E.	β	*p*-Value	95% CI for B
Step 1	Constant	2.48	1.14	-	0.034	0.20; 4.77
	Age	0.07	0.02	0.38	**0.003**	0.03; 0.11
Step 2	Constant	3.17	1.12	-	0.006	0.92; 5.42
	Age	0.04	0.02	0.22	0.104	−0.01; 0.09
	Cardiovascular diseases	1.67	0.64	0.34	**0.012**	0.39; 2.96

Notes: B—non-standardized regression coefficient; S.E.—standard error; β—the standardized regression coefficient; CI—confidence interval. Significant *p*-values are presented in bold. R^2^ = 0.23.

## Data Availability

The original data presented in the study are openly available in Dataverse at https://doi.org/10.48510/FK2/C22JKP.
